# Oesophageal cancer cases recorded in the Zambia National Cancer Registry: a cross-sectional study

**DOI:** 10.11604/pamj.2023.44.128.32907

**Published:** 2023-03-14

**Authors:** Samson Shumba, Patrick Musonda, Dorothy Lombe, Gershom Chongwe, Violet Kayamba

**Affiliations:** 1 University of Zambia School of Public Health, Department of Epidemiology and Biostatistics, Lusaka, Zambia,; 2 Cancer Diseases Hospital, Nationalist Road, Lusaka, Zambia,; 3 Tropical Diseases Research Centre, Ndola, Zambia,; 4 Tropical Gastroenterology and Nutrition Group, University of Zambia School of Medicine, PO Box 50398, Lusaka, Zambia

**Keywords:** Esophageal, cancer, survival, registry

## Abstract

**Introduction:**

the aim of this study was to determine what proportion of patients with confirmed esophageal cancer at the largest hospital in the country were recorded in the Zambia National Cancer Registry (ZNCR).

**Methods:**

we reviewed esophageal cancer records at the University Teaching Hospital (UTH) and ZNCR, between 2015 and 2017. Using Stata version 15, data were summarised and the Kruskal-Wallis was used to compute comparisons, Kaplan-Meier curves for survival estimates and Cox regression for associated factors.

**Results:**

included in the final analysis were records for 222 patients with confirmed esophageal cancer and of these 51/222 (41%) were appearing in the ZNCR. The mean age of the patients was 56.2 years (SD, 13.0) and only 2/222 (1%) were confirmed alive at the time of data analysis. The median time from endoscopic diagnosis to histological confirmation was 12.5 days (IQR 7.5 - 21.5) and arrival at the Cancer Diseases Hospital (CDH) for treatment was 20 days (IQR 10 - 34). The overall median survival time in the study was 259 days (CI 95%; 151 - 501). Age, sex, time to diagnosis, histological classification and grade of tumour did not show any evidence of predicting survival in both the univariate and multivariable cox regression model (p>0.05).

**Conclusion:**

a significant proportion of esophageal cancer cases seen at UTH were not included in the national registry suggesting that official figures for the prevalence of esophageal cancer in Zambia are underestimated. There is an urgent need to improve the collection of data on esophageal cancer in Zambia.

## Introduction

Esophageal cancer is the eighth most common cancer and the sixth leading cause of cancer related deaths globally [1]. In 2020, there were 604,100 new cases and 544,706 deaths attributed to esophageal cancer. There is a marked variation in the occurrence of esophageal cancer globally with adenocarcinoma predominating in high income countries and squamous cell carcinoma in low and middle income countries [1]. High incidence regions for esophageal squamous cell carcinoma include a belt extending through central to south-eastern regions of Asia and the eastern part of sub-Saharan Africa (SSA) [2-4]. Globally, the incidence of esophageal cancer is commoner in males than females [5,6].

In Zambia, esophageal cancer is the fifth most common cancer and it is ranked third among the causes of cancer related deaths [1]. There is evidence of an increasing occurrence of esophageal in Zambia [7] with many patients presenting at a relatively young age [8]. The observation of early onset esophageal cancer has also been reported in other parts of SSA [9]. In addition, many of the cases of esophageal cancer are diagnosed at an advanced stage with very poor patient outcomes due to limited therapeutic options [10,11]. However, data from many SSA countries are estimates as there are limited population-based cancer registries compounded by poor diagnostic facilities and limited overall access to specialised health care.

The Zambia National Cancer Registry (ZNCR) an affiliate of the African Cancer Registry Network was established to collect data on cancer in Zambia [12]. Recently, there have been strides at improving data collection with computerised networks connected to the Cancer Diseases Hospital (CDH) the major cancer treatment institution in Zambia. CDH offers comprehensive cancer treatment including radiotherapy, chemotherapy and surgery. In addition, ZNCR collects data on cancers diagnosed and treated directly from all ten provinces of Zambia. However, it is not clear what proportion of esophageal cancer patients are actually recorded in the ZNCR.

**Study objective:**in this study, the main objective was to evaluate records of patients diagnosed with esophageal cancer at the largest tertiary hospital in Zambia and establish the proportion of records included in the ZNCR. This was done in order to have a clearer estimate of the burden of esophageal cancer in the country, and provide evidence for the need to improve such data collection.

## Methods

**Study design:**this was a cross-sectional study of hospital records, as information on esophageal cancer was obtained as a snap shot. These data were analysed for various factors of interest. For this analysis, we included all available records between 2015 and 2017 inclusive. This was done in order to eliminate any form of bias.

**Study setting:**records were obtained from the University Teaching Hospital (UTH) in Lusaka Zambia, which is the largest referral hospital in the country.

**Population:**records for cases of esophageal cancer during the period under review.

**Data extraction:**data on esophageal cancer diagnosed during endoscopy were collected and records of histological confirmation sought from the hospital´s histopathology laboratory from the beginning of 2015 to the end of 2017. Histological confirmation was reported as recorded on the pathology reports including the sub-type (adenocarcinoma, squamous cell carcinoma, Kaposi´s sarcoma, and neuroendocrine) and grade of the tumour (poorly, moderately and well-differentiated tumours). Records that did not have histopathological confirmation were excluded from the final analysis. All confirmed cancer patients at UTH are referred to the CDH for further management. We therefore evaluated the records at CDH to determine what proportion of these patients were seen at that institution. Data from CDH are then directly fed onto the Zambia National Cancer Registry (ZNCR) database, where the final information on the occurrence of these cancers are collected and stored.

**Study variables:**variables included in the study were as follows: Age, sex, body mass index, marital status, employment status, time to histological diagnosis, time to CDH for treatment, distance to the hospital, tumour sub-type and treatment modality.

**Ethical considerations:**the University of Zambia Biomedical Research Ethics Committee (UNZABREC) reference number 381-2019 and National Health Research Authority (NHRA) approved this study.

**Data analysis of quantitative variables:**for descriptive purposes, frequencies and percentages were computed for categorical variables whereas; continuous variables were checked for normality using Shapiro Wilk and reporting was done using mean (standard deviation) and median (interquartile range). Where appropriate, a t-test, ranksum or Kruskal Wallis test was used to determine the association of continuous variables, while a Chi-square or Fishers exact test was used for categorical variables. To describe survival, the Kaplan Meier graphs were used. Log rank test was used to check for the equality of the functions. Cox regression models were carried out to investigate the effects of predictors on survival of patients. Goodness of fit involved the use of the Akaike Information Criteria (AIC) and Bayesian Information Criteria (BIC). All tests p-value < 0.05 was considered significant. Missing data were not included in comparative analyses.

## Results

**Figure 1:**
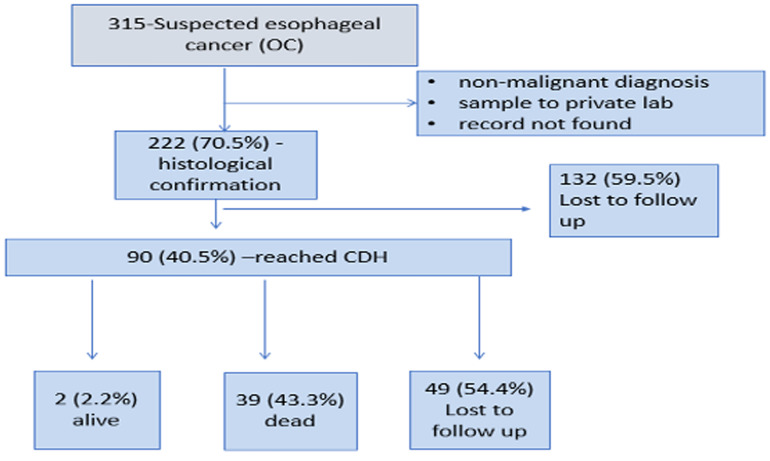
records that were retrieved and information gathered and included in this study

**Proportion of esophageal cancer cases captured in the ZNCR:**endoscopic records for 315 patients suspected with esophageal cancer were retrieved with 222/315 (71%) of them having histological confirmation. The flow of cases evaluated is as indicated in Figure 1.

The mean age for esophageal cancer patients was 56 years (SD = 13.0). Nineteen percent (43/222) of the patients were below the age of 45 years. The median body mass index was low at 18.2 (IQR 16.2 - 19.7) kg/m^2^, Table 1. Evaluation of records at the Cancer Diseases Hospital revealed that 51/222 (41%) of these patients were seen for therapy and thus recorded in the Zambia National Cancer Registry (ZNCR), Table 1.

**Table 1:** background characteristics of esophageal cancer patients and the characterisation of their tumours

Characteristics	Esophageal Cancer N (%) n=222
**Age;** mean (SD¹), years	56.2(13.0)
Female	68(31.0)
Male	151(69.0)
Never married	10(17.2)
Married	42(72.4)
Widowed	5(8.6)
Separated	1(1.7)
Unemployed	23(35.9)
Employed	41(64.1)
Time from endoscopy to histological confirmation of esophageal cancer in days; median (IQR)	12.5(7.5-21.5)
Time from endoscopy to Cancer Diseases Hospital in days; median (IQR^2^)	20(10 - 34)
BMI^3^; median (IQR) kg/m^2^	18.2(16.2 - 19.7)
Distance from residence to hospital; median (IQR) in km	13.5(8.8 - 438.3)
Poorly Differentiated tumour	18(18.0)
Moderately Differentiated tumour	64(64.0)
Well Differentiated tumour	18(18.0)
Chemotherapy	19 (46.3)
Radiation	9 (22.0)
Chemotherapy and Radiation	13 (31.7)
¹Standard Deviation, ^2^Interquartile Range, ^3^Body Mass Index	

**Figure 2:**
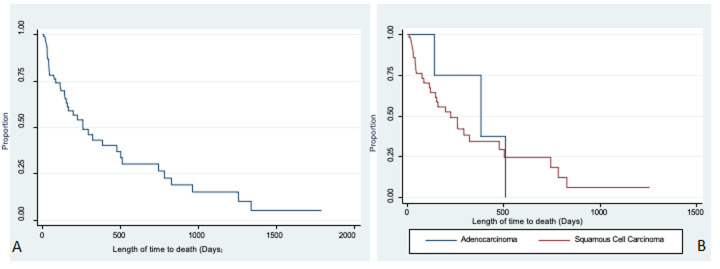
Kaplan-Meier curves, A) survival in days of oesophageal cancer patients; B) survival in days stratified by histological type

**Length of time from endoscopic case detection to the final outcomes:**at the time of data analysis, only 2/222 (1%) of the esophageal cancer patients were confirmed alive, and still being actively followed up at CDH. The remaining patients were either confirmed dead 39/222 (17.6%) or lost to follow-up. It took a median time of 12.5 days (IQR 7.5-21.5 days) from endoscopic detection of the cancers to histopathological diagnosis. Similarly, it took a median of 6 days (IQR 2-16 days) after histological confirmation for patients to arrive at CDH for initial treatment consultation. Therefore, median time from initial endoscopic detection of the cancer to CDH consultation was 20 days (IQR 10 - 34). Figure 2A shows Kaplan Meier survival estimates of patients with a median survival of 259 days (CI 95%; 151 - 501). The results also show that the median time for squamous cell carcinoma (SCC) was 223 days (CI 95%, 118 - 475) compared to 383 days for adenocarcinoma (Figure 2B). There was no evidence to suggest a difference in survival by histological classification, (log rank, p=0.55).

**Factors associated with esophageal cancer survival:**the mean age of patients still alive or lost to follow-up was 55.5 years (SD = 12.8) compared to 59.3 years (SD = 13.8) of those confirmed dead, a difference that was statistically significant, p= 0.049. Median time from endoscopic diagnosis to histological confirmation was 13 days (IQR 8-23 days) for a surrogate of those confirmed alive patients and lost to follow-up compared to 11 days (IQR 7-16 days) days for those who died, p=0.14. Time to diagnosis, age, sex, grade of tumour and histological classification were investigated in predicting survival of patients diagnosed with esophageal cancer. In both the univariate and multivariable Cox regression, there was no evidence that any of the variables under investigation were predicting survival of esophageal cancer patients. Controlling for all other factors (age, diagnosis and histological classification of cancer), one-day increase in time to diagnosis of patient´s increased the risk of death by a factor of 1.01 (CI 95%, 0.11 - 0.89; p= 0.73), however we could not rule out chance finding. The findings in the study suggest no evidence of a difference in the hazard of death between patients with SCC and adenocarcinoma (HR, 1.72; CI 95% 0.31 - 9.44; p= 0.24) (Table 2).

**Table 2:** estimates from survival analysis of patients with esophageal cancer and gastric cancer (univariate and multivariable Cox regression)

Predictors	HRc (CI 95%)	P-value	HRa (CI 95%)	P value
**Age**	1.01(0.98-1.03)	0.50	1.00 (0.96-1.05)	0.84
**Time to Diagnosis**	0.98 (0.94-1.01)	0.16	1.01 (0.95-1.07)	0.73
**Sex**				
Female	1 (Ref)		1 (Ref)	
Male	0.64 (0.33-1.23)	0.18	0.43 (0.12-1.57)	0.20
**Grade**				
Poorly Differentiated	1 (Ref)		1 (Ref)	
Moderately Differentiated	5.36 (0.66-43.68)	0.12	4.52 (0.53-38.36)	0.17
Well Differentiated	5.15 (0.55-47.97)	0.15	9.22 (0.83-101.97)	0.07
**Histology**				
Adenocarcinoma	1 (Ref)		1 (Ref)	
Squamous cell carcinoma	1.44 (0.43-4.76)	0.55	1.72 (0.31-9.44)	0.24

## Discussion

In this study, we present evidence that formal figures of the occurrence of esophageal cancer in Zambia are underestimated. We found that close to half of the confirmed cases of esophageal cancer were not included in the national cancer registry, a source of data for international country estimates. Therefore, our study has shown that the burden of esophageal cancer in Zambia is underestimated. We have also shown that the median survival for esophageal cancer patients in Zambia is very low.

The exact prevalence of esophageal cancer in many sub-Saharan African countries is not known. Many countries rely on estimates using data obtained in hospital records. However, such sources of data are limited, because they do not capture cases that never reach hospitals. In the Zambian scenario, the high number of esophageal cancer cases being missed by the cancer registry is alarming. The country´s meagre resources available for healthcare and research are directed toward diseases that are perceived to have high prevalence and less towards those thought to be rare such as esophageal cancer. This study has brought to light the fact that there are more cases of esophageal than reported, and that more resources will need to be channelled to this deadly disease.

It is known that in many instances, esophageal cancer is diagnosed late and our previous work on gastric cancer identified delays in endoscopy referral as one of the main contributing factors [13]. This audit has similarly shown that the major source of delay is before the initial case detection. It has also revealed an even worse patient outcome than reported earlier. Early case detection is difficult when the disease does not have specific early warning symptoms. Esophageal cancer patients therefore do not know that they are developing this potentially lethal malignancy until it has advanced and they already have obstructive symptoms. In many countries, there are no screening programmes and detection of early disease is therefore compromised [14,15].

Our data have also shown that the source of delay was not in acquisition of histopathology reports or initiation of therapy. The number of patients who actually arrived at CDH for treatment was low with many of them having either died or were lost to follow-up. It would not be unreasonable to assume that the lost to follow-up patients had poor outcomes, as esophageal cancer does not resolve spontaneously and there were no other cancer treatment centres in Zambia. Poor survival figures are not unique to Zambia as they are low in many developed and developing countries. For example, in Europe the 1- and 5-year survival rates were reported as 33.4% (95% CI 32.9-33.9%) and 9.8% (95% CI 9.4-10.1%), respectively [16]. In our study, only 1% of the patients were still alive and being followed up at CDH. This is an alarming statistic. We do acknowledge, that the proportion of patients lost to follow-up was high but it is unlikely that these miraculously got better, but rather more likely that their outcomes were poor. However, there is need to carry out a prospective follow-up of these patients in order to get a more accurate picture of what is going on.

As expected, our results showed that squamous cell carcinoma was the most common histological sub-type. However, neither histological sub-type nor the tumour grading was associated with patient outcomes. We had no information on the staging of these cancers, as that required radiological examination such as computerised tomography or endoscopic ultrasound which were not readily available to most of the patients due to cost and infrastructural barriers. On analysis of factors related to patient outcomes using Cox regression, we found no evidence that any of the demographic and clinical factors were associated with patient survival. Similarly other investigators reported demographic characteristics that were not associated with esophageal cancer [17].

With this information, we propose the following strategies; (1) that factors hindering early referral for endoscopy be systematically reviewed in Zambia, (2) recording of patient data with a diagnosis of esophageal cancer be improved at UTH by collecting data directly form the endoscopy unit, (3) computerised data bases be linked between the endoscopy unit, histopathology laboratory, the CDH and ZNCR (4) an active programme for patients who are lost to follow-up be instituted (5) Prospective studies to obtain a full picture of the predictors of survival be instituted and lastly (6) efforts aimed at finding early detection strategies be intensified.

**Limitations:**this is a retrospective analysis of patient records. Despite the availability of well-kept endoscopy and histopathology records at the UTH and treatment records at CDH, there were still some missing data. We were unable to collect information on patients who were lost to follow-up and therefore did not have sufficient power to predict survival regardless of their relevance. The other potential limitation in the study was selection bias due to cost hindrances. Patients who could not afford to pay user fees and transport costs may have not been able to come to hospital and their data subsequently left out.

## Conclusion

Esophageal cancer cases reported in the ZNCR are an underestimation of the true occurrence of the disease. There is need to improve data collection, to enable more accurate representation of the burden of disease. Patient outcomes are also very poor with a high proportion of individuals lost to follow-up.

### What is known about this topic

- *Esophageal cancer is a growing health problem in the east and southern parts of Africa;*
- *There is a paucity of population-based cancer registries in sub-Saharan Africa;*
- *Available data on esophageal cancer from sub-Saharan Africa are estimates*.

### What this study adds

- *Esophageal cancer data in the Zambia Cancer Registry are under represent the true burden;*
- *Many esophageal cancer patients are lost to follow-up and do not get treatment;*
- *Once esophageal cancer is diagnosed, prompt therapy is available to those who present to the Cancer Diseases Hospital in Lusaka*.
